# Bone marrow mesenchymal stromal cells for diabetes therapy: touch, fuse, and fix?

**DOI:** 10.1186/s13287-022-03028-2

**Published:** 2022-07-26

**Authors:** Zahra Azizi, Roya Abbaszadeh, Roxana Sahebnasagh, Amir Norouzy, Elahe Motevaseli, Kathrin Maedler

**Affiliations:** 1 Department of Molecular Medicine, School of Advanced Technologies in Medicine, Tehran University of Medical Sciences, No. 88, Italia St, Keshavarz Blvd., Tehran, Iran; 2 Department of Biology, Philipps-University Marburg, Marburg, Germany; 3 Department of Energy & Environmental Biotechnology, National Institute of Genetic Engineering and Biotechnology (NIGEB), Tehran, Iran; 4 Islet Biology Laboratory, Centre for Biomolecular Interactions Bremen, University of Bremen,, Leobener Straße 5, NW2, 28359 Bremen, Germany

**Keywords:** MSC, BM-MSC, Fusion, Diabetes, Regeneration, Beta-cell, Insulin, Bone marrow stem cells

## Abstract

Bone marrow mesenchymal stromal cells (BM-MSCs) have anti-inflammatory and pro-survival properties. Naturally, they do not express human leukocyte antigen class II surface antigens and have immunosuppressive capabilities. Together with their relatively easy accessibility and expansion, they are an attractive tool for organ support in transplantation and regenerative therapy. Autologous BM-MSC transplantation alone or together with transplanted islets improves β-cell function, graft survival, and glycemic control in diabetes. Albeit MSCs’ capacity to transdifferentiate into β-cell is limited, their protective effects are mediated mainly by paracrine mechanisms through BM-MSCs circulating through the body. Direct cell–cell contact and spontaneous fusion of BM-MSCs with injured cells, although at a very low rate, are further mechanisms of their supportive effect and for tissue regeneration. Diabetes is a disease of long-term chronic inflammation and cell therapy requires stable, highly functional cells. Several tools and protocols have been developed by mimicking natural fusion events to induce and accelerate fusion in vitro to promote β-cell-specific gene expression in fused cells. BM-MSC-islet fusion before transplantation may be a strategy for long-term islet survival and improved function. This review discusses the cell-protective and anti-inflammatory characteristics of BM-MSCs to boost highly functional insulin-producing cells in vitro and in vivo, and the efficacy of their fusion with β-cells as a path to promote β-cell regeneration.

## Background

For the first time in 1966, Friedenstein et al. introduced and characterized bone marrow-derived cells from mice and called a group of fibroblast–shaped cells in culture with the potential to differentiate into multilineages in vitro “bone marrow mesenchymal stromal cells (BM-MSCs)” [1]. Later studies found these mesenchymal stromal/stem cells (MSCs) in many other tissues such as blood, umbilical cord blood, amniotic fluid, skin, foreskin, heart muscle, lung, pancreas, adipose tissue, dental pulp [2–4], and also in human islets [5]. BM-MSCs are multipotent cells that can be isolated from bone marrow aspiration and easily expand in culture without loss of function. Besides their capacity to self–renew in vivo [6, 7], they can differentiate into several cell types of mesenchymal, endodermal, and ectodermal origins [8]. In 2006, the international society for cellular therapy introduced minimal characterization criteria for MSCs, i.e., the expression of surface antigens CD73 (identified by the MAb SH3 and SH4), CD90 and CD105 (identified by the MAb SH2) and the negativity for CD34 (primitive hematopoietic progenitors marker), CD45 (pan–leukocyte marker), CD19 and CD79a (B cell marker), CD14 and CD11b (monocyte and macrophage marker) and HLA class II as well as their adherence to plastic dishes in culture and their differentiation capacity into chondrogenic, osteogenic, and adipogenic lineages [9]. The bone marrow only consists of approximately 0.01–0.001% MSCs [10]. However, BM-MSCs rapidly proliferate in culture, and their proliferation rate accelerates in platelet lysate instead of fetal bovine serum (FBS) [11] and at lower oxygen tension as mimicry of their native microenvironment [12]. Injected BM-MSCs into blastocyst can proliferate and differentiate into all organs in response to tissue-specific signals [8]. MSCs, despite their high proliferative potential, are negative for OCT4, preventing teratoma formation [13].

MSCs circulate through the bloodstream, migrate, and home in on injured tissues. They have not only the potential to transdifferentiate into different lineages [14, 15] but can also fuse with somatic cells in vitro as well as in vivo [16–18]. Transplantation of MSCs into injured tissue improves repair mechanisms [19–21] through modulation of the immune response [22], transdifferentiation [23, 24], fusion with target cells in injured tissues [21] and increased proliferation [23].

The lack of donor antigens, low level of HLA class I, and absence of HLA class II make allogeneic MSCs a suitable source for transplantation [10, 25]. Therefore, BM-MSCs are in trials for the therapy of autoimmune diseases, e.g., graft versus host disease (GVHD) [26], Crohn’s disease [27], multiple sclerosis [28], and type 1 diabetes [29–31].

In summary, multiple cell regeneration supportive properties make MSCs a suitable tool for clinical studies through their

- Easy accessibility
- Immunomodulatory, anti-inflammatory, and anti-apoptotic effects
- Angiogenic potential
- Capability to differentiate into multilineages like adipocytes, neurons, and pancreatic β-cells
- Lack of teratoma formation.

Many studies show MSCs derived from adipose tissue and umbilical cord as promising sources for therapy (please see previous comprehensive overviews [32–37]). This review focuses specifically on multipotent bone marrow mesenchymal stromal cells (BM-MSCs), underlying mechanisms how they can protect pancreatic β-cells, their potential use for β-cell regeneration and as source for cell-based diabetes therapy. Furthermore, studies of spontaneous and induced BM-MSCs-target cell fusion as method for cellular differentiation are discussed. Fusion of MSCs with β-cells to make β-MSCs have been performed exclusively using BM-MSCs. We hypothesize that fusion of MSCs with target cells shares similar mechanisms independently of the source of MSCs.

### BM-MSCs in clinical studies for diabetes therapy

Over 50 clinical trials for MSC applications, reaching from tackling diabetes-related vascular damage and impaired wound healing to treating new-onset type 1 diabetes (T1D) and type 2 diabetes (T2D), have been registered in the last decade [30, 38]. The first trial of autologous BM-MSCs transplantation for T1D has started in 2010 at Uppsala University Hospital (NCT01068951); 20 adult patients with newly diagnosed T1D received combined BM-MSCs/insulin or insulin only, which resulted in a preserved or even increased C-peptide response in BM-MSC-treated patients without any discernible side effects during the 1-year study period. However, there was no difference in HbA1c levels or insulin doses [29]. In T2D, several studies show improved C-peptide and lowered insulin requirements by BM-MSCs therapy [39, 40]. A recent meta-analysis which included bone marrow-, umbilical cord-, adipose-, and Wharton’s jelly-derived MSC therapy in patients with T1D and T2D confirmed improved C-peptide levels, lower HbA1c and blood glucose levels, and lesser insulin requirement without serious or chronic adverse responses [38] and concludes a more beneficial effect of MSCs in the treatment of T1D than in T2D. In contrast, another recent meta-analysis [41] presented no difference in C-peptide levels in T1D but improvement in T2D and comes to the conclusion of MSC therapy being more effective in improving β-cell function in T2D.

Inclusion of several underpowered studies, different study designs, trial durations, heterogenous patient groups (e.g., age and time of diagnosis), non-transparent data reports from several trials and the use of MSCs of various origins raise some doubts on reproducibility of various studies and their conclusions. Therefore, well-designed standardized randomized studies with larger numbers of patients and longer observation periods are needed to finally prove a long-term effective MSC therapy for patients with T1D and T2D [30, 38].

### MSCs in diabetes: mechanisms of action

The clinical application of MSCs is motivated from in vitro cell culture and in vivo studies in animal models of numerous diseases with promising results [28, 30, 42, 43]. BM-MSCs can differentiate into functional β-cells [3, 44]; (Tables 1, 2), but also only little if any transdifferentiation capacity of MSCs-to-β-cells [45–47] and no spontaneous cell–cell fusion [24, 48] have been observed.

**Table 1:** Examples of secreted factors from BM-MSCs with effects on immune, vascular, and β-cells

Target cells	Paracrine factors released by BM-MSCs
T cells, B cells	IDO, PGE2, NO, TGF-β, HGF
Dendritic cells	IL-6, IL-10 (interleukin-6,10)
Natural killer cells	HLA-G5 (Human Leukocyte G isoform)
Neutrophils, monocytes	HO1 (heme oxygenase-1) [50, 52, 54]
Endothelial cells	
Smooth muscle cells	VEGF, MCP-1, bFGF
Hematopoietic stem/progenitor cells	TGF-β1, IL-8, HGF, PIGF (placental growth factor) [105]
β-cells/islets	CXCL12/SDF-1 [58], Annexin A1 [65]
Progenitor cells	HGF, IL-IRa^+^MSCs [71]

**Table 2:** Examples of expressed β-cell/endocrine transcription factors and functional markers after spontaneous and induced MSC-to-β-cell transdifferentiation or after β-MSC fusion

Studies type	MSC to β-cell	Transcription factors/function markers
In vitro	By chemicals/gene transfer	Insulin, glucagon, somatostatin, pancreatic polypeptide, Glut2, Foxa2, Pdx1, Ngn3, Nkx2.2 [3, 44]
	Cell fusion	Increased
		Neurod1, Nkx2.2, MafA, Pdx1
		Insulin, glucagon negative [78]
In vivo	Spontaneous differentiation	Insulin, glucagon, somatostatin, Pdx1, Glut2, Nkx2.2, and Nkx6.1 [24, 48]

**Fig. 1:**
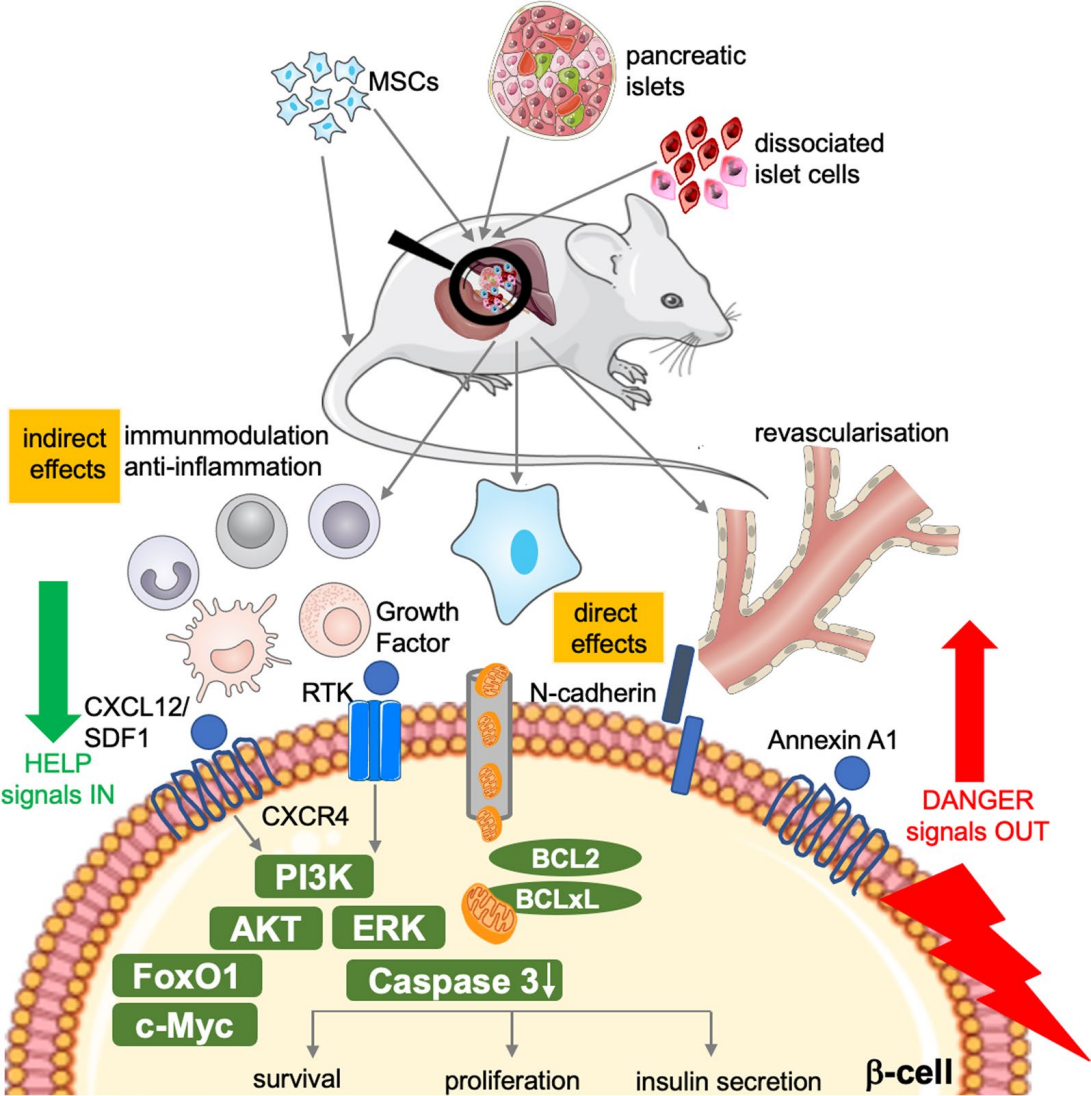
Protective mechanisms of MSCs in vivo. Both sole transplantation and co-transplantation of MSCs with islets or dissociated islet cells into the portal vein or the kidney capsule affect β-cell mass replenishment and transplantation outcome through indirect (immune and endothelial cells) and direct effects between MSCs and the β-cell. Danger signals sent out from the β-cell are detected by MSCs and tissue repair mechanisms are in place, e.g., via CXCL12/SDF signals, growth factor signals, N-cadherin-mediated direct cell–cell contacts, and secreted annexin, or by the formation of tunneling nanotubes which enable the exchange of mitochondria. In concert, these mechanisms promote β-cell survival/apoptosis protection, proliferation, and improved β-cell function

MSCs regulate both innate and adaptive immune responses by coordinating cell-to-cell contacts and the secretion of soluble humoral factors with immunosuppressive effects on target cells, such as immune, vascular, and endocrine cells [22]. Such factors show supportive anti-inflammatory, anti-apoptotic, and pro-survival effects on pancreatic β-cells [31, 49] (Fig. 1, Table 1).

BM-MSCs reduce activation, survival, and proliferation of CD4^+^ or CD8^+^ cytotoxic T cells; they were shown to diminish activation of dendritic and natural killer (NK) cells, increasing Tregs and reducing cytokine secretion [22, 50, 51], thus balancing the immune response. Thereby, the lesser T cell proliferation and the induction of regulatory Tregs can simultaneously occur in a monocyte-dependent manner [52] with the outcome of decreased inflammation [53]. All these effects would be of great importance toward inhibiting autoimmunity and T1D progression. An inflammatory environment propels MSCs to respond with anti-inflammatory effects to promote rapid tissue regeneration. Activated MSCs diminish pro-inflammatory signals mainly by releasing soluble factors: (1) secreting Prostaglandin E2 (PGE2) and Indolamine-2,3-dioxygenase (IDO) in response to pro-inflammatory insults, such as lipopolysaccharide, TNF-α and nitric oxide (NO) secreted from active macrophages and injured tissues, (2) producing TNF-stimulated gene-6 protein (TSG-6) that interacts with pro-inflammatory macrophages to reduce NF-κB signaling cascades and (3) promoting expression and secretion of anti-inflammatory Interleukin-1 receptor antagonist (IL-1Ra) [54] with highly protective effects on β-cell function and survival [55, 56]. MSCs also secrete several other factors with immunomodulatory properties on β-cells, e.g., transforming growth factor-beta (TGF-β), hepatocyte growth factor (HGF), interleukin-10 (IL-10) [57], heme oxygenase-1 (HO-1) [14] and matrix metalloproteinases-2 and -9 (MMP-2 and MMP-9) [15, 29] (Table 1).

BM-MSC-released SDF1/CXCL12 bound to CXCR4 on β-cells improve their survival via activation of AKT [58]. Both in vitro and in vivo studies showed that BM-MSCs activate important pro-survival AKT, ERK, and FoxO1, all leading to the expansion of the β-cell population [59–62]. Additionally, activation of the major downstream executor of the apoptotic pathway, caspase 3, is reduced in β-cells when islets are co-transplanted with MSCs [63]. MSCs can also reduce endoplasmic reticulum (ER) stress-induced apoptosis in islets through downregulation of ER chaperone (BIP) and apoptosis-inducing ER stress protein (CHOP) and enhanced c-Myc expression (64). The functional improvement in MSC-islet co-cultures partially comes from annexin A1 which is secreted and highly expressed in MSCs. The direct exposure of high concentrations of secreted ANXA1 during MSC-islet co-culture adds to the paracrine mediated supportive effects [65].

Also, direct cell–cell contacts of MSCs and islet cells after co-transplantation contribute to the β-cell-protective effect, shown through N-cadherin interactions which improves insulin secretion efficiently [66]. Mitochondria from MSCs can transfer through tunneling nanotube-like structures to β-cells, a response probably induced through “danger signals” sent from damaged islets during transplantation stress to MSCs, which then support survival and insulin secretory function [67] (Fig. 1).

MSCs express a set of chemokine receptors such as CX3C chemokine receptor 1 (CX3CR1) and CXC chemokine receptor 12 (CXCR12), which are attracted by their specific ligands CX3CL1 and CXCL12 expressed in pancreatic islets [68]. Hence, MSCs could also serve as vehicle for effective drug, gene, or protein delivery to targeted cells, i.e., to islet cells in vivo [69, 70]. Such gene transfers could promote insulin production and vascularization and prevent apoptosis in islets [71]. For example, co-transplantation of MSCs overexpressing hepatocyte growth factor (HGF) and interleukin-1 receptor agonist (IL-1Ra) (HGF^+^IL-IRa^+^MSCs) in islets improves the outcome of islet transplantation [70]. Also, PDX1-expressing MSCs can transform into β-like cells which produce insulin after transplantation in vivo, display glucose-stimulated insulin secretion, and reduce hyperglycemia in diabetic mice [72].

BM-MSCs have been widely used for autologous, allogeneic transplantation or co-transplantation, to reduce the severity of disease and injuries [73]. Initially, autologous bone marrow-derived stromal progenitor cells were infused to facilitate engraftment and contribute to the recovery of hematopoiesis after bone erosion and bone marrow transplantation for cancer treatment [74]. BM-MSCs have been progressively explored as adjuvants to improve the outcome of therapies and avoid relapse. For instance, BM-MSCs increase body weight in weak and severely diabetic STZ (streptozotocin)-induced mice [73], together with the restoration of normoglycemia and β-cell function. Co-transplantation of MSCs improves and prolongs transplantation efficiency and outcome [68], again through paracrine effects which suppress immune response and inflammation [63, 75–77] (Fig. 1).

### BM-MSCs for β-cell regeneration

BM-MSCs promote physiological tissue repair by migrating into injury sites, such as in cardiac infarction, where systemically as well as locally transplanted MSCs home in on injured tissue. As diabetes is a chronic disease with subclinical levels of inflammation rather than an acute tissue injury, spontaneous migration of endogenous MSCs may be limited. However, GFP-labeled infused BM-MSCs localize near pancreatic ducts, possibly to accelerate differentiation of pancreatic progenitor cells in addition to promote immune regulation and functional improvement in β-cells within islets [59]. BM-MSC-promoted β-cell regeneration has been observed indirectly by proliferation induction of pancreatic ductal cells and islet cell clusters, which led to increased pancreatic progenitor cell numbers and insulin production [23]. Successful induction of cell fusion, in vivo or in vitro, maybe a strategy to combine favorable characteristics of BM-MSC with the powerful glucose metabolism regulation by highly functional β-cells (Fig. 3) [78].

### Cell fusion as tool for cell regeneration

Life begins with cell fusion. During fertilization, sperm fuses with the egg. Later, fusion repeatedly happens during organ development and in adults, e.g., macrophage fusion with target cells during infection or MSC fusion with damaged cells for tissue repair [79].

For the first time, spontaneous cell fusion resulting in multinucleated cells in vitro was seen in 1927 by W. H. Lewis [80]. Later in 1960, fusion of two heteroploid mouse cells resulted in the generation of hybrid cell lines from two different sarcomas [81]. Then, Harris and Ringertz successfully fused various cell types from different species and discovered that UV-inactivated Sendai virus induces fusion in cell culture. By fusing mammalian Hela cells with chicken erythrocytes, they observed condensed and inactive chromosomes in birds’ erythrocytes, but interspecies heterokaryons express chicken-specific RNA, indicating that DNA and RNA synthesis had been re-activated in the chicken nuclei after fusion [82]. Constant co-culture of two different mouse cell lines results in approximately 10% hybrid cells after three months, according to chromosome variations due to spontaneous fusion [81]. Fusion of mouse BM-MSCs with other cell types in culture showed that fused cells adopt the phenotype of the recipient cells, such as beating cardiac myocytes [83].

That BM-MSCs can transdifferentiate both directly and indirectly after fusion with injured cells in vivo is supported by a previous study: While a sub-population of BM-MSCs within a mixed population of injured epithelial cells transdifferentiate into epithelial cells, fusion is another phenomenon in up to 1% of the cells identified as epithelial^+^polyploid cells [57].

**Fig. 2:**
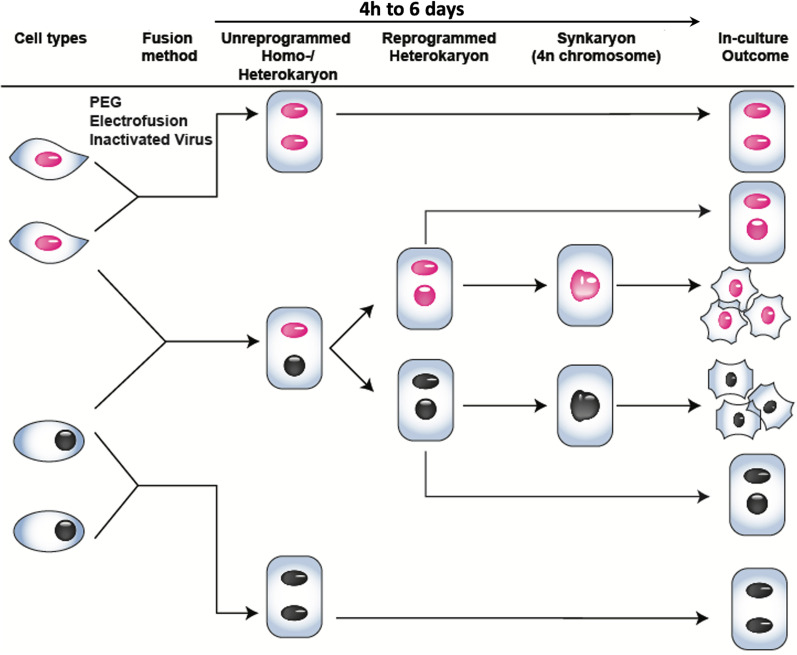
Outcome of cell fusion in culture. Fusion is a natural phenomenon that can occur between cells of the same or of different types. In vitro cell fusion can be achieved through three major methods, i.e., chemicals including polyethylene glycol (PEG), inactivated viruses, and electric pulse (electrofusion). At the beginning, a cell is formed in which the nuclei do not merge (heterokaryon). This state could be transient in case both cells are from the same species which ends up in a synkaryon, or they remain separated in interspecies heterokaryons. Chromatin remodeling starts within few hours after heterokaryon formation and its combination with the genetic reprograms will define the fate of the resultant synkaryon cell up to the development of hybrid cells

Cell fusion is a powerful attempt toward tissue regeneration. Three fundamental methods to induce it in culture have been developed using (1) inactivated viruses, (2) chemical agents including PEG, and (3) electric pulses [82, 84, 85]. Fusion occurs between the same or different cell types and results in transient homo- or heterokaryons with three distinct outcomes: homokaryons (> 1 of the same nuclei in a polyploid cell), heterokaryons (> 1 of different nuclei in a polyploid cell), and synkaryons (nuclei have fused in a polyploid cell) (Fig. 2). While homo- or heterokaryons are non-dividing cells and frequently transient, the nucleus of a synkaryon has a combined chromosome pool of all nuclei; they can become proliferative and eventually make hybrids. During cell fusion, the epigenetic and genetic information of the different cell types is combined. This leads to new cellular expression patterns, starting within few hours of the heterokaryon state by remodeling the chromatin and switching-on transacting regulators at key loci [79, 86]. Fusion between interspecies cells, like rat and human cells, often leads to the formation of stable interspecies heterokaryons, where nuclei are not combined. This phenomenon brings a unique opportunity to trace the variation of the chromosome pool in an intact nucleus after the fusion event and to study the first phases and epigenetic changes in lineage reprogramming [87], in iPSC generations [86, 88] and differentiation [89]. The cell fate could be bidirectional after fusion; but is later fixed to only one of the parental cell fates, depending on the relative nuclear dosage [90]. Fusion of a pluripotent with a somatic cell gives rise to a dominant pluripotent cell fate [91]. The dominant fate after fusion of two different somatic cells depends on their cell type, e.g., heterokaryons from human keratinocytes and mouse myotubes display a more keratinocyte fate [90]; from mouse melanoma and rat hepatoma results in both melanin and albumin production, but not at the same time [92]. By increasing the ratio of somatic to pluripotent stem cells, one can activate the expression of all cell lineage determinants sequentially [93]. The new cell fate in heterokaryons is determined by the structural information in the chromosomes, such as DNA methylation/demethylation patterns, transcription factors, and non-coding RNAs leading to repression or activation of specific genes; dominant and recessive fates are changeable after fusion depending on the activity of chromatin-modifying enzymes [94].

### BM-MSCs and cell fusion in vivo

BM-MSCs can turn into a new phenotype via fusion with target cells in vivo [17, 57, 79, 95, 96]. BM-MSCs traced by the Cre-loxP system in mice after transplantation show transdifferentiation into cardiac, lymphatic and kidney tissues. Surprisingly, cell fusion is the ubiquitous phenomenon. Monitoring these cells over 5 months did not reveal any signs of cancer development [96]. The ratio of spontaneous fusion increases with age and time after transplantation [97] and the frequency of fusion is higher with larger cells with larger cytoplasmic volume, e.g., Purkinje neurons, skeletal myotubes, cardiomyocytes, and hepatocytes [79]. One must still accept that spontaneous cell fusion in vivo without the presence of pro-inflammatory conditions or intense tissue injury is a rare event [57, 79, 98].

First evidence of in vivo reprogramming after fusion was based on changes in the chromatin as sign of a fused cell: BM-MSCs injection into mice leads to BM-MSCs-purkinje-neuron-binucleated-heterokaryons in the mouse brain [97]. First time reported in humans in a cerebellar tissue autopsy from a female recipient of male MSCs shows 0.1% of her Purkinje neurons tetraploid (XXXX) or containing both X and Y chromosomes (XXXY) as a sign of cell fusion of male BM-MSCs and female neurons [97]. Similar results come from pancreas autopsies from opposite-gender cord blood MSCs recipients, which detected nuclear fusion based on two sets of opposite sex chromosomes in one nucleus. 1.5% of opposite-sex insulin-expressing cells were seen and 0.76% of the insulin-producing cells are polyploid with three or even more sex chromosomes and enlarged nuclei, assuming that at least half of the differentiation events resulted from cell fusion in the pancreas, even in a non-diabetic environment. From other studies, there is lesser evidence of transdifferentiation via cell fusion to β-cells in vivo in humans [99] or mice [24]. As functional heterokaryons do exist in vivo [97, 100, 101], it remains to be assessed whether these polyploid cells seen after BM-MSC transplantation will progress to fully functional cells.

In line with the hypothesis that tissue damage is a prerequisite for MSC trafficking and fusion, other studies observed an increase in heterokaryon cells after apoptosis induction [18] or chronic inflammation [98]. Therefore, various methods were established to improve fusion efficiency in vitro [82, 84, 85], e.g., a microfluidic device which improves fusion efficiency in suspension culture [102].

### BM-MSCs-β-cell fusion: evidence for highly functional β-MSCs

Compared to other differentiation methods, e.g., gene transfer, in vitro cell fusion imitates nature as a phenomenon that occurs to rescue cells in danger and has a well-documented safety profile in numerous clinical studies. Improving cell fusion in vitro can be a strategy for transplantation to restore survival of cells in damaged tissue. As a proof of concept, Flatt et al. established a functional human β-cell line (1.1B4) through electrofusion of the epithelial-like human pancreatic carcinoma cell line (PANC–1) with human pancreatic islet cells. 1.1B4 hybrid cells have stable characteristics in culture and secrete insulin upon glucose stimulation. The implantation of 1.1B4 cells into Streptozotocin (STZ)-diabetic mice decreases glucose levels and improves diabetes [89]. However, recently, several 1.1B4 clones have been found inhomogeneous as they contain a mixture of rodent and human cells and do not fully retain the human β-cell phenotype [103].

Given the protective nature of BM-MSC-islet co-transplantation and the feasibility of cell fusion, Yanai et al*.* produced β-MSCs from rat BM-MSCs and mouse dispersed islet cells by electrofusion. They showed slightly higher insulin secretion after one day in β-MSCs which dramatically increases compared to co-cultured BM-MSCs/dispersed islets after 20 days, with higher proliferation rates and lower caspase 3 expression levels [104].

**Fig. 3:**
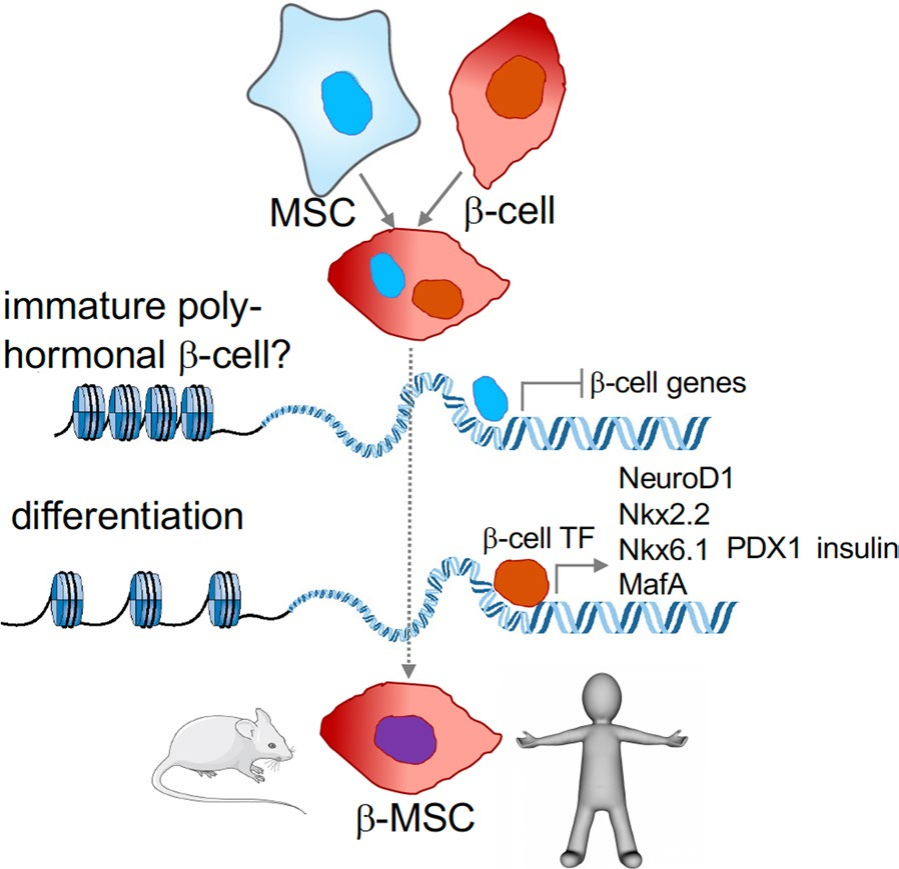
MSCs and β-cells and fusion to β-MSCs. β-MSC transdifferentiation after fusion may initially result in immature polyhormonal cells and later process to the expression of mature β-cell markers. Epigenetic modifiers and transcription factors are major contributors to this process inside the shared cytoplasm. Further differentiation may be achieved after transplantation

Later, we developed and improved a fusion protocol of human BM-MSCs with rat INS1E β-cells, which led to 5% to up to 30% successfully fused cells seen as β-MSC heterokaryons. Fusion showed an increase in β-cell functionality and identity genes, compared to INS1E control cells, such as insulin, NKX6.1, Nkx2.2, Neurod1, MafA, and Pdx1. Such induction of functional genes after fusion was also confirmed upon fusion of BM-MSCs with human-dispersed islet cells from organ donors. Importantly, fused cells show improved functionality over co-cultured MSC-dispersed islets [78] (Fig. 3). In vivo β-MSC-islets also gradually normalized blood glucose levels after transplantation [104].

Despite promising results, cell fusion is still in its experimental stage and many questions on the characteristics of newly fused cells and how to steer the fusion process remain unanswered. None of the previous studies compared numbers of heterokaryons and hybrids that were formed in culture. Long-term stability and functionality of newly formed islets in vivo and to what extend MSCs remain mature in β-MSC-islets has never been investigated.

When somatic cells fuse with pluripotent cells, most of the resulting hybrids maintain their pluripotent character, and only few hybrids switch to somatic types. Our studies showed higher fusion rates by increasing the number of β-cells over BM-MSCs. Whether higher fusion rates correlate with the number of insulin positive β-MSCs and how fused cells choose to transdifferentiate into their desirable fate has not been addressed. In-depth investigation and characterization of interspecies heterokaryon stages will help answering these questions and evaluate quantity and quality of fusion and the genetic profile and stability of fused cells before they are ready to enter clinical trials.

## Conclusion

BM-MSCs are a natural physiological source for β-cell protection after injury and can be fused with β-cells to obtain reprogrammed functional β-MSCs with improved survival. In vivo studies confirm their stability and glucose-normalizing efficacy over weeks. However, long-term in vivo studies and the characterization of genomic and epigenetic patterns of insulin-producing β-MSCs are needed to prove safety, stability, and potential for diabetes therapy. Nature’s imitation of spontaneous in vivo fusion gives hope in regenerative medicine as another strategy for cell differentiation, including β-cells.

## Data Availability

Not applicable. No specific data have been generated for this review.
